# Unveiling the pharmacological potential of *Coelogyne suaveolens*: An investigation of its diverse pharmacological activities by in vivo and computational studies

**DOI:** 10.1002/fsn3.3867

**Published:** 2023-12-07

**Authors:** Taslima Akter Eva, Husnum Mamurat, Md. Habibul Hasan Rahat, S. M. Moazzem Hossen

**Affiliations:** ^1^ Department of Pharmacy, Faculty of Biological Science University of Chittagong Chittagong Bangladesh

**Keywords:** analgesic, anxiolytic, *Coelogyne suaveolens*, computational work, GCMS, sedative activity

## Abstract

The medicinal potential of *Coelogyne suaveolens*, a traditional medicinal plant, was investigated through in vivo and molecular docking studies. The ethyl acetate fraction of the plant's acetonic extract was subjected to various bioactivity tests to assess its analgesic, anxiolytic, and sedative effects on Swiss albino mice. Furthermore, we used GCMS to identify the bioactive chemicals in the extract's ethyl acetate fraction. The root and bulb extracts demonstrated significant analgesic activity in acetic acid‐induced writhing, hot plate, and tail immersion tests in a dose‐dependent manner when compared to the control. Again, the extract exhibited moderate anxiolytic activity in the elevated plus maze test at a dosage of 400 mg/kg body weight, while the root extract showed significant anxiolytic activity in the hole board test at the same dosage. Significant sedative activity was observed in the hole cross, open field, and rotarod tests at a dosage of 400 mg/kg. According to molecular docking studies, the extract has the potential to serve as an analgesic medication by reducing the enzymatic activity of cyclooxygenases 1 and 2. Overall, the findings suggest that *C. suaveolens* has substantial therapeutic potential for the development of novel treatments for pain, anxiety, and sleep disorders.

## Graphical abstract

The study explored the medicinal properties of *Coelogyne suaveolens* through in vivo experiments and molecular docking. The plant extract exhibited analgesic, anxiolytic, and sedative effects in mice with identified bioactive compounds. Molecular docking suggested its potential as an analgesic targeting cyclooxygenases, highlighting its therapeutic potential for pain, anxiety, and sleep disorders.

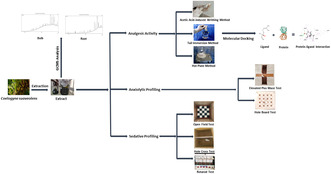

## 1 INTRODUCTION

Natural medications have been used for centuries to heal complex ailments without side effects and at affordable prices. Different varieties of naturally occurring chemical compounds in medicinal plants contribute to their therapeutic qualities. With the development of scientific studies researchers have been able to successfully discover many phytochemical components in medicinal plants and accelerate these findings by using in silico studies. These chemicals have been shown to exhibit a variety of physiological actions and are utilized for preventative measures (Sofowora, 1996). Due to a lack of scientific facts, mainstream drugs have made experts distrust the safety of traditional medicine. In the nineteenth century, humans isolated medicinal plant active ingredients and invented quinine using Cinchona bark (Phillipson, 2001). The results of these studies convinced medical researchers to put their faith in alternative medicine and pursue it further. Additionally, it has begun to gain popularity among the public as an alternative to conventional medication because of its higher efficacy, lower risk of health complications, and lower cost. Many powerful conventional medicinal agents, such as vincristine, paclitaxel, and vinblastine (Cragg & Newman, 2005), cardioprotective drugs like digoxin (Morris et al., 2006), narcotic analgesics such as morphine (Rates, 2001), and anti‐malarial drugs such as artemisinin and quinine (Queiroz et al., 2009), were first discovered as a result of the influence of phytochemistry. Studies have been conducted recently on a variety of medicinal plants, including *Hedychium spicatum*, whose rhizomes are used to treat pain, diarrhea, nausea, liver issues, vomiting, inflammation, headache, stomachaches, and fever, as well as *Erigeron bonariensis*, which is said to have antioxidant, antibacterial, and anti‐inflammatory properties, and *Paederia foetida*, which has anti‐diabetic, anti‐hyperlipidemic, antioxidant, nephro‐protective, anti‐inflammatory, antinociceptive, antitussive, thrombolytic, anti‐diarrhoeal, sedative‐anxiolytic, anti‐ulcer, hepatoprotective, and anthelmintic activity (Mahanur et al., 2023; Sarma et al., 2023; Singh et al., 2023). On the other hand, in silico methods, as described in recent research (Hossain et al., 2023; Madden et al., 2020), create predictions about numerous characteristics of chemical compounds, particularly ADME traits, along with their biological function and toxicity, using the current data and molecular composition information. These models are based on the basic concept that a chemical's molecular structure encodes both its inherent characteristics and its potential interactions. As a result, QSAR (quantitative structure–activity relationship) models can be constructed, enabling chemical response predictions based purely on structural data.

Pain is a mental, physical, and unpleasant phenomenon that is acknowledged as a worldwide health issue (Kumar Paliwal et al., 2016). Most pain in the body begins with some sort of illness or injury (Mills et al., 2019), where analgesics, a class of drug, are used to treat pain by blocking different pathways (Van Rensburg & Reuter, 2019). Nonsteroidal anti‐inflammatory medications (NSAIDs) and opioids are the most commonly utilized analgesics nowadays (Rosenblum et al., 2008). NSAIDs typically work by inhibiting the formation of prostaglandins via the blocking of an enzyme called cyclooxygenase (Gunaydin & Bilge, 2018). NSAIDs have been especially helpful in the treatment of inflammation and pain; however, they are well known to be related to gastrointestinal injury. Selective Cyclooxygenase‐2 inhibitors have been shown to reduce the risk of digestive tract issues, but they have also been linked to adverse cardiovascular events (Zarghi & Arfaei, 2011). Serious NSAID‐related problems, including bleeding, perforation, and mortality, occur at a yearly estimated rate of around 2% for NSAID consumers (De Cosmo & Congedo, 2015). On the other hand, opioids are known to promote addiction, physical dependency, and tolerance. When people discontinue opioids after a lengthy period of use, they develop bodily (e.g., muscle cramps, diarrhea, and anxiety) and psychological symptoms of depression (Hijazi et al., 2017). In addition to physical pain, the world is affected by several other serious conditions, such as anxiety disorders, which rank as the most frequent mental illness in the United States (7.3%). Anxiety disorders (especially GAD and panic disorder) frequently overlap with depressive illness, which complicates treatments (Bandelow & Michaelis, 2015). Among different drug options, bupropion's ability to inhibit norepinephrine and dopamine reuptake makes it a potent antidepressant with fewer side effects (Friesner et al., 2004). Drugs like MAOIs and tricyclic antidepressants (TCAs) are commonly used in therapy because of their ability to inhibit the increase of monoamine metabolism and decrease reuptake capability. Many of the standard treatments for depression, however, come with serious drawbacks. Therefore, new antidepressant medicines are necessary to avoid these consequences (Nasrin et al., 2022).

Orchids are renowned for their aesthetic appeal, and they are frequently utilized as decorative items in households, offices, and public spaces. While most individuals appreciate their beauty, others have discovered practical applications for them. For a long time, people from various regions of the world have employed orchids for medicinal purposes. However, the use of orchids in medicine has decreased over time due to insufficient research to determine their efficacy and adverse effects. The Chinese were the first to discover the medicinal properties of orchids, and they continue to use them for medicinal purposes today, mainly in the form of medicinal tea. Dendrobium, in particular, is believed to have several medicinal properties, including antioxidant (Luo et al., 2016), hypoglycemic (Pan et al., 2014), immune modulatory (He et al., 2016), cardioprotective (Dou et al., 2016), hepatoprotective, and antitumor (Tang et al., 2017). To advocate for the traditional functions of orchids, *Coelogyne suaveolens*, a member of the family *Orchidaceae* native to central China, Assam, the eastern Himalayas, and Thailand, has been selected. The central question addressed by this research is whether *C. suaveolens*, a traditional medicinal plant, possesses therapeutic properties that can be scientifically validated. In comparison to previously published literature on medicinal orchids, this research offers a unique contribution by focusing on *C. suaveolens*, which has received limited attention in prior studies. While orchids, especially Dendrobium species, have been investigated for their medicinal properties, including antioxidant, hypoglycemic, immune modulatory, cardioprotective, hepatoprotective, and antitumor effects, this study distinguishes itself by delving into the potential therapeutic benefits of *C. suaveolens*.

As no prior research exists on the analgesic, anxiolytic, sedative activities of this orchid, the purpose of this research is to assess its bioactive phytochemicals and their pharmacological actions. This research addresses a specific research gap in the field of medicinal plant‐based therapies by providing empirical evidence for the therapeutic potential of *C. suaveolens*. It fills the gap between traditional medicinal knowledge and modern scientific validation, demonstrating its effectiveness in pain relief, anxiety reduction, and sleep disorders, paving the way for the development of novel treatments in these areas.

## 2 MATERIALS AND METHODS

### 2.1 Chemicals

Aceclofenac, diazepam, and morphine were provided by United Chemicals & Pharmaceuticals Limited, Chittagong, Bangladesh. The Department of Pharmacy, Faculty of Biological Science, University of Chittagong provided other analytical‐grade reagents.

### 2.2 Collection and identification of the plant

In 2021, with the help of a well‐known local traditional healer, the bulbs and roots of the matured plant were collected, which was newly identified in Bangladesh (Huda et al., 2021). Then it was approved by a noted taxonomist, Dr. KamrulHuda and Assistant horticulturist Mr. Md. Owahidul Alam from department of Botany, University of Chittagong under herbarium no. Dpcu/2021/011.

### 2.3 Crude extract preparation

The study materials (bulb and root) were washed, sliced into tiny pieces, and dried in the sunlight for seven days in a semi‐shed. The plant components were processed into a powder using a mechanical grinder after drying. The bulb and root of the plant were powdered and immersed in acetone. After 13 days of intermittent stirring, the filtrate was concentrated after the solution was filtered using a rotary evaporator by evaporation under reduced pressure and below 50°C (Stu‐art, UK).

### 2.4 Solvent–solvent partitioning

Using ethyl acetate solvent, crude acetone extracts of the bulb and root of *C. suaveolens* underwent solvent–solvent partitioning using the technique developed by VanWagenen et al. (1993).

### 2.5 GC MS analysis

A mass spectrometer from Agilent Technologies (Santa Clara, CA, USA) was used to analyze the chemical constituents of *C. suaveolens* extract using a 7890A capillary gas chromatography system. For the analysis, approximately 6 μL of crude extract (1% w/v) was diluted with methanol:chloroform:water (2.5:1:1) and injected into a fused silica capillary column (HP‐5MSI, 90 m × 0.25 mm) coated with a 0.25 μm film consisting of 95% dimethyl‐poly‐siloxane and 5% phenyl. Helium (99.999%) was used as the carrier gas, flowing at a rate of 1 mL/min. The mass chromatogram was analyzed with the MS quad at 150°C and the source temperatures at 250°C, respectively. The National Institute of Standards and Technology (NIST) mass spectrometry data center serves as a reference for identifying the chemical components of the extracts by comparing their MS spectra to the NIST database (Al‐Nuri et al., 2022).

### 2.6 Experimental animals

We acquired Swiss albino mice from the BCSIR laboratory in Chittagong for our experiment. These mice were of both sexes, aged 4–5 weeks, and had a weight ranging from 20 to 25 grams. These mice were housed in clean, dry polypropylene cages within the Animal House of the Department of Pharmacy at the University of Chittagong. They were subjected to a 12‐hour light–dark cycle at a temperature of 25 ± 2°C and a relative humidity level ranging from 45% to 55%. Throughout the duration of the investigation, the mice were provided with a nutritionally adequate diet and a free water supply. Twelve hours prior to and throughout the duration of the trial, food was not given. The Departmental Ethical Review Committee of the University of Chittagong's Department of Pharmacy granted approval for the clinical animal experiment under consent number Pharm/CUDP‐16, 2022:08. At the completion of each experiment, all mice were sacrificed under diethyl ether anesthesia.

### 2.7 Study design for in vivo testing

During each evaluation, six groups of mice, with five mice in each group, were used for each investigation. For analgesic activity, Group (I) served as the control (1% tween‐80 10 mL/kg), Group (II) served as the standard (Diclofenac sodium 50 mg/kg was used in the acetic acid writhing study, and morphine sulfate 10 mg/kg was used for tail immersion and the hot plate method), and Groups (III) and (IV) received bulb extract at 200 and 400 mg/kg, respectively, and Groups (V) and (VI) received root extract at 200 and 400 mg/kg, respectively. For anxiolytic and sedative activity, Group (I) served as the control (1% tween‐80 10 mL/kg), Group (II) served as the standard (Diazepam 1 mg/kg was used in both the anxiolytic and sedative tests), and Groups (III)–(VI) received the bulb and root extract at doses of 200 and 400 mg/kg, like in the previous manner.

### 2.8 Acute toxicity study

The previously outlined approach was used to conduct an acute toxicity investigation. Each group consists of five mice that overnight fasted before receiving the extract. Each animal group received oral dosages of 1000, 2000, 3000, and 4000 mg/kg of body weight for each extract. After receiving the plant extract, they were not given food for an additional 3–4 h. Each animal was observed for thirty minutes, twenty‐four hours, and then three days. At least once per day, the mice were examined for any changes in their epidermis, fur, mucous membrane, eyes, respiration rate, circulation rate, and central and autonomic nervous systems. One‐tenth of the median lethal dose would be the effective dose (LD50) (Afrin et al., 2021).

### 2.9 Investigation of analgesic activity

#### 2.9.1 Acetic acid‐induced writhing test

The acetic acid‐induced writhing test was a behavioral observation and measurement approach that revealed painful stimulation in mice. This research was conducted using the approach developed by Koster, with some adjustments made by Dambisya and Lee (Dambisya et al., 1999; Koster et al., 1959). After giving the standard and extract to the mice, 0.7% glacial acetic acid (10 mL/kg) was administered intraperitoneally (IP) 15 min after giving the standard and 30 min after giving the extract. This caused pain that showed up as constrictions or writhing of the abdomen. 5 min later, each mouse in each group was carefully watched for 20 min to count how many times it had writhed. Diethyl ether was used to euthanize the treated mice after each observation. The percent inhibition of abdominal writhing was used to measure the level of analgesia. This was done with the following formula:

$$\text{Percent Inhibition of Pain}=\left(\mathrm{Nc}-\mathrm{Nt}\right)/\mathrm{Nc},$$

where *Nc* denotes the number of writhings in the control group and *Nt* denotes the number of writhing's in the treatment group.

#### 2.9.2 Tail immersion method

The methodology created by Di Stasi et al. was used in this study. Using this thermal approach, the central analgesic activity of the extracts under investigation was assessed (di Stasi et al., 1988). Before the 30 min of treatment, about 2–3 cm of each mouse's tail was put into a water bath with warm water kept at 50 ± 1°C, and the time it needed for the mouse to pull its tail out of the warm water was marked down. The animals that flicked their tails within 3–5 s were chosen for this research. To keep the tail from getting hurt, a cut‐off time of 15 s was chosen. After the initial test, the mice that were given the drug were checked at 30, 60, 90, and 120 min (Malairajan et al., 2006). Wrapping the animals carefully immobilized them so that measurements could be taken. The following formula was used to determine what proportion of the maximum possible effect (% MPE) was achieved (Fan et al., 2014):

$$\begin{array}{c} \%\text{of}\;\mathrm{MPE}=\left(\;\text{Post drug Latency}–\mathrm{Pre}\;\text{drug Latency}\right)/\\ \left(\mathrm{Cut}\;\mathrm{off}\;\text{time}–\mathrm{Pre}\;\text{drug Latency}\right)\times 100. \end{array}$$

The percentage of time that the tail immersion elongated compared to the control time was measured. The central analgesic effect will be stronger if the percentage of elongation period is higher.

The following equation was used to get the percentage of time elongation (Kumawat et al., 2012):

$$\begin{array}{c} \%\text{of Elongation}=\left(\text{Latency of Treatment}–\text{Latency of Control}\right)/\\ \text{Latency of Treatment}\times 100 \end{array}$$

#### 2.9.3 Hot plate method

In this investigation, a commonly accessible Eddy's hot plate with an electrical heating surface maintained at 55–56°C was used. The methodology by Arunkumar et al was followed with a slight modification (Arunkumar et al., 2019). Animals in each group were subjected to the hot plate individually; reaction times were recorded as the number of seconds it took for the animals to lick their forepaws or jump in response; and the reaction strengths of individual mice were measured at 30, 60, and 120 min intervals before and after drug treatment. To prevent injury to the paws, a 15‐second cutoff time was instituted. The following formula was used to detect the % increase in reaction time:

$$\%\text{Increase in reaction time}=\frac{\left(\text{post treatment}-\mathrm{pre}\;\text{treatment}\right)\;}{\mathrm{pre}\;\text{treatment}}\times 100$$

### 2.10 Anxiolytic profiling

#### 2.10.1 Elevated plus maze method

The elevated plus maze is a crucial tool for studying the neuroprotective and anxiolytic effects of test medications (Sunanda et al., 2014). Since rodent's fear heights and prefer confined areas, they spend a disproportionate amount of time there. When animals enter an open‐arms environment, they often get frightened and are unable to move (Pellow et al., 1985). The main benefits of this test approach are (a) its speed, simplicity, and reduced testing time; (b) the absence of the need for unpleasant stimuli or training; and (c) its predictability and reliability in assessing the anti‐anxiety and anti‐anxiety medication properties (Emamghoreishi et al., 2005; Vogel et al., 2010). The elevated plus maze is a plus‐shaped tool 40 cm above the ground, featuring two open arms (25 × 5 cm) and two closed arms (25 × 5 cm, 16 cm long) perpendicular to each other [Miyakawa et al., 1996]. Each test subject was positioned in the center of the maze, oriented toward an open arm, and the stopwatch was initiated. Over a 5‐minute period, several factors were observed. Initially, the mice exhibited a preference for either the open or closed arms. The count of entries into both open and closed arms was noted, with an entry defined as all four paws of the mouse being within an arm. Afterwards, the animals received various substances, including a control group receiving saline, a standard group administered with diazepam, and multiple test samples of *C. suaveolens*. After a 30‐minute interval from treatment, each animal was placed back in the maze's center. For a final 5‐minute period, the duration that each animal spent in both the open and closed arms was documented.

#### 2.10.2 Hole‐board test

Hole‐board equipment is an installation with holes in the floor that an animal can insert its head into. This is called “head‐dipping.” (File & Wardill, 1975). The length and regularity of head dipping can be used to measure neophilia, which means “directed exploration.” This shows that the animal's ability to move around is independent (Ljungberg & Ungerstedt, 1976). An increased level of head dipping is usually a sign of neophilia, while low levels are often caused by a lack of neophilia or show that the animals get more anxious (Crawley, 1985). Hence, the anxiety level rises as head‐dipping is reduced and lowers when the opposite occurs (Kliethermes & Crabbe, 2006). Mice were placed individually on the hole board device for 30 min before being administered with control, standard, and test samples. We then used a tally counter to measure the number of times each mouse dipped its head into a hole at eye level over a 5‐minute trial session.

### 2.11 Sedative profiling

#### 2.11.1 Open‐field method

The equipment used for this test had a floor space of roughly 0.5 m^2^ and was enclosed by a wall that was 50 cm in height (Gupta et al., 1971). The ground is covered in a pattern of tiny squares that alternate between being white and black in color. The count of squares explored by the mice was documented at intervals of 0, 30, 60, 90, and 120 min post‐oral treatment. This involved the control group (saline), the standard group (diazepam), and varying doses of test samples (200 and 400 mg/kg). A tally counter was used to track the mice's movements within a 3‐minute timeframe.

### 2.12 Hole cross method

This test was conducted in a wooden‐walled chamber (30 cm × 20 cm × 14 cm) without a ceiling. In the middle of the room, a fixed wooden frame divides it into two parts. The wooden barrier had a round hole that was 3.5 cm in diameter and 7.5 cm high. At 0, 30, 90, and 120 min, the number of times the mice passed through the opening between the two chambers was recorded using a tally counter for a period of 3 min (Hossen et al., 2022).

### 2.13 Rotarod method

A rotarod device was used to evaluate the impact on motor coordination; it featured a plant‐shaped base and a 3 cm diameter by 30 cm long iron rod that had a non‐slippery surface. The rod had four disks, dividing it into five equal parts. In a training lesson 24 h before the test, the animals were selected based on their ability to stay on the bar (at 12 rpm) for 2 min. Then, five mice were put on the rod and walked at 12 rpm at the same time, and this was watched for 30, 60, and 90 min. The performance time was automatically recorded as the time between when the animal was put on the spinning bar and when it fell off. The amount of time the animals spent in the machine was tracked for 5 min (300 s) (Doukkali et al., 2015).

### 2.14 In silico study

#### 2.14.1 Ligand preparation

Seven major compounds were chosen from the GC–MS profiling Tables 2a and 2b for a molecular docking analysis to further assess analgesic efficacy. These included alpha‐linolenic acid, {1,6‐Octadien‐3‐ol, 3,7‐dimethyl‐}, isopulegol, geranyl acetate, {bicyclo[3.1.1]heptan‐3‐one, 2,6,6‐trimethyl‐, (1.alpha.,2.beta.,5.alpha.)‐}, {3,6‐dimethyl‐2,3,3a,4,5,7a‐hexahydrobenzofuran}, and {9,12‐octadecadienoic acid (Z,Z)‐}. All of the compounds were obtained in SDF format from the PubChem database. Then, using Open Babel (version 2.3.1), the structures were exported to the pdb format. AutoDock Tools (version 1.5.6)'s ligand preparation module was used to convert these pdb files into pdbqt format.

#### 2.14.2 Protein preparation

Three‐dimensional structures of cyclooxygenase‐1 (PDB ID: 5wbe) (Cingolani et al., 2017) and human cyclooxygenase‐2 (PDB ID: 5ikq) (Orlando & Malkowski, 2016) were downloaded in pdb format from the protein data bank (Berman et al., 2000) for the assessment of analgesic activity. Discovery Studio 2020 was used to make both of the protein structures ready by eliminating water molecules and complex co‐structures. The proteins subsequently went through an energy minimization procedure utilizing the steepest descent and conjugate gradient methods. Energy calculations were done in vacuo using the GROMOS 96 43B1 parameters with the implementation of Swiss‐PDB Viewer (Version 4.1.0). The pdb format was transformed to the pdbqt format employing AutoDock Tools (version 1.5.6), and the final macromolecules have been conserved in this format.

#### 2.14.3 Molecular docking simulation

Molecular docking analyses were carried out via AutodockVina (version 1.1.2) to determine how the compounds selected might work to prevent analgesia by blocking COX‐1 and COX‐2 enzymes. AutodockVina's grid box was maintained at 66.9216, 75.6914, and 64.8721 angstroms (X, Y, and Z) for the cyclooxygenase‐1 and 77.1175, 62.5380, and 57.5579 angstroms (X, Y, and Z) for the human cyclooxygenase‐2. The shell script given by the AutoDockVina developers was used to implement AutodockVina, and the binding affinity of the ligands was measured in terms of kcal/mol (Kumar et al., 2023 Trott & Olson, 2009).

## 3 STATISTICAL ANALYSIS

The data were presented in the form of a mean ± SEM (standard error of mean). “Statistical Package for the Social Sciences” (SPSS, Version 16.0, IBM Corporation, New York) was used for statistical analysis, and a one‐way ANOVA followed by a post hoc Dunnett's test for comparisons was performed. Significance levels were determined as follows: *p < .05, **p < .01, and ***p < .001, indicating statistical significance when compared to the control group.

## 4 RESULTS

### 4.1 Acute toxicity study

There was no indication of any negative consequences in the mice, including reduced motor function, agitation, seizures, diarrhea, coma, or lacrimation, at any of the doses tested. At the concentrations utilized in the tests, no mice died. As a result, the LD50 was confirmed to be greater than 4000 mg/kg.

### 4.2 Phytochemical investigation

#### 4.2.1 Qualitative screening

This study was conducted to verify the presence or absence of preliminary phytochemicals. A variety of phytochemicals, including alkaloids, flavonoids, saponins, tannins, reducing sugars, phenolic compounds, proteins, amino acids, phytosterols, and terpenoids were detected in the *C. suaveolens* bulb and root extracts upon phytochemical screening, as demonstrated in Table 1.

**TABLE 1:** Phytochemical analysis of the ethyl acetate fraction of *C. suaveolens* bulb and root extracts.

Phytochemicals	Bulb	Root
Alkaloids	−	−
Flavonoids	+	+
Saponins	+	+
Tannins	+	+
Phenolic compounds	−	+
Glycosides	+	+
Carbohydrates	−	−
Reducing sugar	−	−
Protein and amino acid	−	+
Phytosterol	+	+
Terpenoids	+	−

### 4.3 GC–MS profiling

**FIGURE 1:**
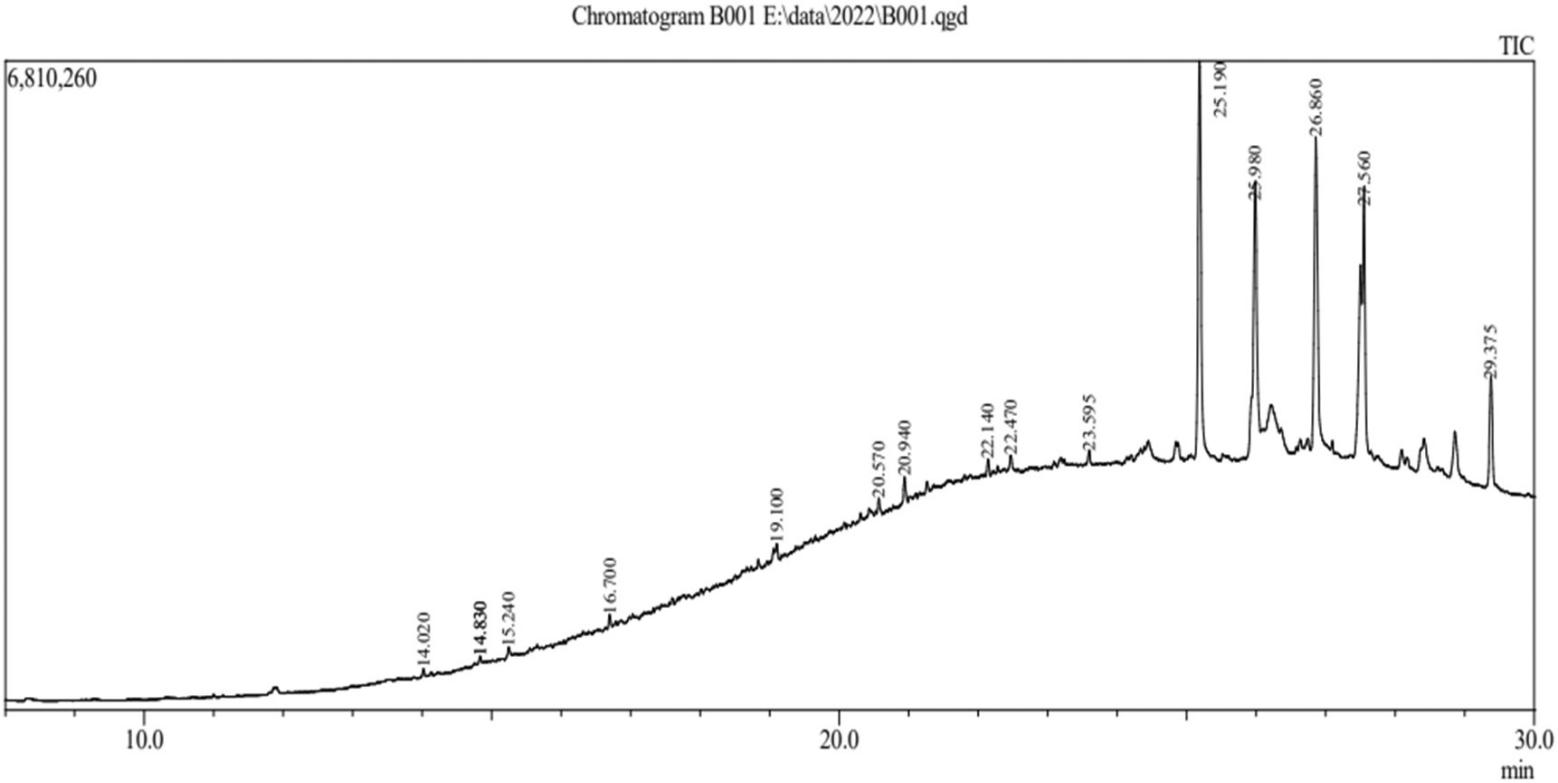
*Coelogyne suaveolens* ethyl acetate fraction's GC–MS chromatogram (Bulb).

**FIGURE 2:**
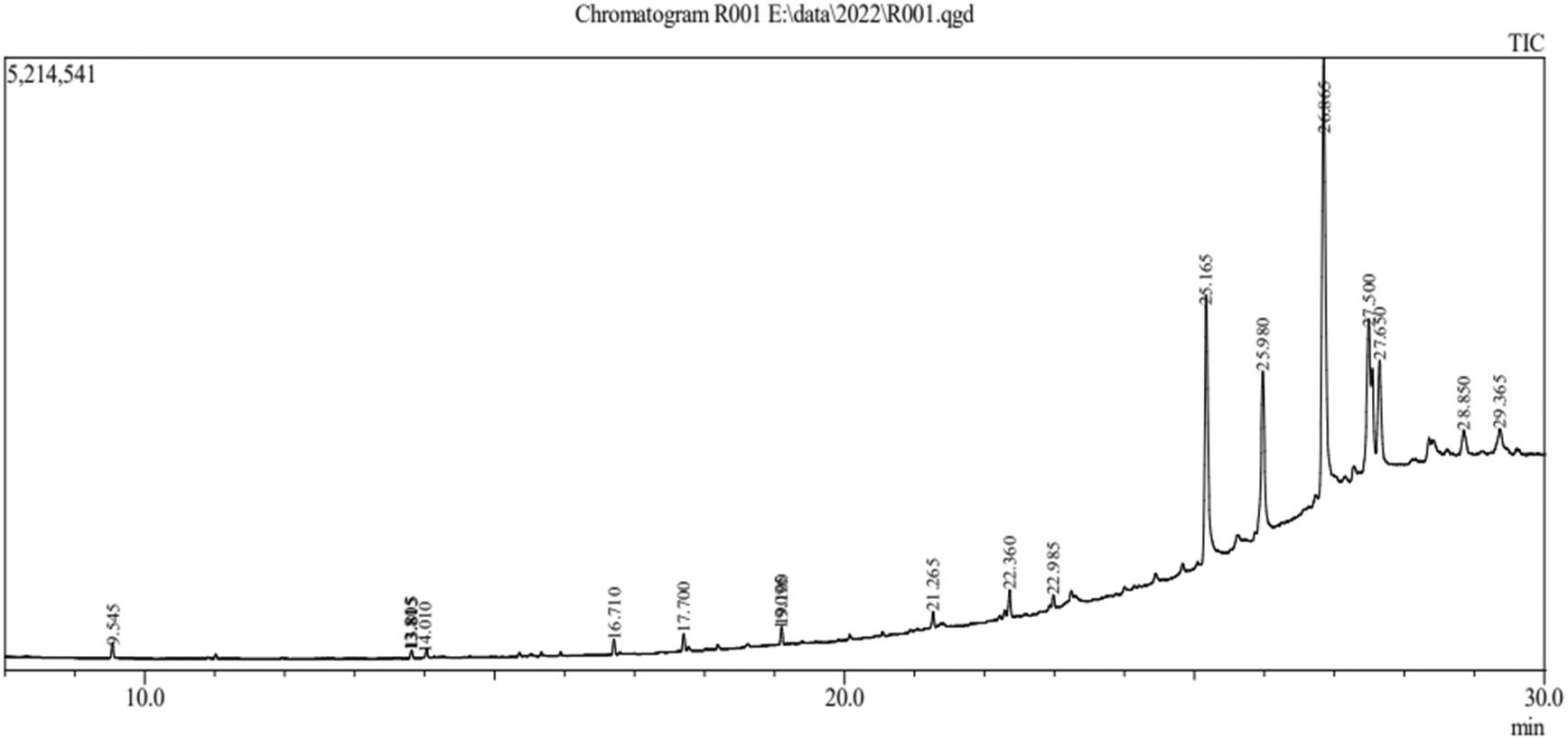
*Coelogyne suaveolens* ethyl acetate fraction's GC–MS chromatogram (Root).

The phytoconstituent‐rich ethyl acetate fraction of the acetonic extract of *C. suaveolens* bulb and root was subjected to GC–MS analysis (gas chromatography–mass spectrometry). A total of 16 compounds were identified from the bulb extract, and an additional 16 compounds were found in the root extract, each exhibiting diverse phytochemical activities. Figures 1 and 2 show the chromatogram, and Tables 2a and 2b list the chemical components and their molecular formula, molecular weight (MW), retention time (RT), and concentration (%).

**TABLE 2a:** Compounds identified by GCMS analysis in the bulb of *Coelogyne suaveolens.*

SL No	Compound name	Molecular formula	Molecular weight (g/mol)	RT (min)	Concentration (%)
1	Naphthalene, decahydro‐1‐pentadecyl‐	C_25_H_48_	348.6	25.978	16.634
2	(2,6,6‐Trimethylcyclohex‐1‐enylmethanesulfonyl)benzene	C_16_H_22_O_2_S	278.4	25.186	35.540
3	4,4′‐Dimethoxy‐2,2′‐dimethylbiphenyl	C_16_H_18_O_2_	242.31	26.861	29.340
4	2‐Methyltetracosane	C_25_H_52_	352.7	27.555	9.793
5	7‐Hexadecenal, (Z)‐	C_16_H_30_O	238.41	29.381	4.847
6	.alpha.‐Linolenic acid, TMS derivative	C_21_H_38_O_2_Si	350.6	36.110	0.833
7	Undec‐10‐ynoic acid, decyl ester	C_21_H_38_O_2_	322.5	14.017	0.169
8	[1,1′‐Bicyclopropyl]‐2‐octanoic acid, 2′‐hexyl‐, methyl ester	C_21_H_38_O_2_	322.5	14.833	0.445
9	9,12‐Tetradecadien‐1‐ol, acetate, (Z,E)‐	C_16_H_28_O_2_	252.39	15.246	0.207
10	E,E,Z‐1,3,12‐Nonadecatriene‐5,14‐diol	C_19_H_34_O_2_	294.5	16.697	0.210
11	(R)‐(−)‐14‐Methyl‐8‐hexadecyn‐1‐ol	C_17_H_32_O	252.4	19.100	0.297
12	2‐Octylcyclopropene‐1‐heptanol	C_18_H_34_O	266.5	20.520	0.096
13	Ethanol, 2‐(9,12‐octadecadienyloxy)‐, (Z,Z)‐	C_20_H_38_O_2_	310.5	20.941	0.259
14	2‐(4‐Hydroxybutyl)cyclohexanol	C_10_H_20_O_2_	172.26	22.132	0.138
15	7‐Hexadecyn‐1‐ol	C_16_H_30_O	238.41	22.475	0.148
16	(2R,3R,4aR,5S,8aS)‐2‐Hydroxy‐4a,5‐dimethyl‐3‐(prop‐1‐en‐2‐yl)octahydronaphthalen‐1(2H)‐one	C_15_H_24_O_2_	236.35	34.891	0.556

**TABLE 2b:** Compounds identified by GCMS analysis in the root of *Coelogyne suaveolens.*

SL No	Compound name	Molecular formula	Molecular weight (g/mol)	RT (min)	Concentration (%)
1	1,6‐Octadien‐3‐ol, 3,7‐dimethyl‐	C_10_H_18_O	154.25	9.537	0.477
2	Geranyl Acetate	C_12_H_20_O_2_	196.29	13.812	0.492
3	7‐Tetradecene	C_14_H_28_	196.37	14.027	0.204
4	1‐Eicosanol	C_20_H_42_O	298.5	16.705	0.294
5	Bicyclo[3.1.1]heptan‐3‐one, 2,6,6‐trimethyl‐, (1.alpha.,2.beta.,5.alpha.)‐	C_10_H_16_O	152.23	17.702	0.882
6	7‐Hexadecenal, (Z)‐	C_16_H_30_O	238.41	19.103	0.349
7	9,12‐Octadecadienoic acid (Z,Z)‐	C_18_H_32_O_2_	280.4	22.359	0.381
8	Isopulegol	C_10_H_18_O	154.25	22.988	0.180
9	3,6‐Dimethyl‐2,3,3a,4,5,7a‐hexahydrobenzofuran	C_10_H_16_O	152.23	25.171	23.096
10	Androstan‐17‐one, oxime, (5.alpha.)‐	C_19_H_31_NO	289.5	25.969	8.130
11	4‐Biphenyltrimethylsiloxane	C_15_H_18_OSi	242.39	26.849	41.098
12	5.beta.‐Androstan‐17‐one, 3.alpha.‐(trimethylsiloxy)‐, O‐methyloxime	C_23_H_41_NO_2_Si	391.7	27.493	7.633
13	Cyclobarbital	C_12_H_16_N_2_O_3_	236.27	28.838	0.385
14	Diethofencarb	C_14_H_21_NO_4_	267.32	30.278	7.903
15	Tetraconazole	C_13_H_11_Cl_2_F_4_N_3_O	372.14	29.309	0.046
16	3‐Chloropropionic acid, heptadecyl ester	C_20_H_39_ClO_2_	347	19.103	0.349

### 4.4 In vivo analgesic activity

#### 4.4.1 Acetic acid‐induced writhing method

In this procedure, the examined extracts administered at 200 and 400 mg per body weight exhibited a reduction in writhing occurrences among rodents when compared to the control group. Notably, the bulb and root extract at the 400 mg/kg dose displayed a marked reduction in writhing incidents (indicated by a significant *p*‐value of less than .001). This substantial decrease in writhing pointed to their enhanced efficacy as peripheral analgesic agents, as detailed in Table 3. Additionally, when comparing the effects of bulb and root extracts, the bulb extract displayed the highest percentage of writhing inhibition at both the 200 and 400 mg/kg doses, surpassing the performance of the root extract.

**TABLE 3:** Evaluation of the analgesic effect of *Coelogyne suaveolens* bulb and root extracts by the acetic acid‐induced writhing method.

Treatment	Writhing count					Mean ± SEM	% Inhibition
	M‐1	M‐2	M‐3	M‐4	M‐5		
Control	57	32	45	34	56	45 **±** 5.15	0
Standard	6	6	7	3	5	5.4 **±** 0.68***	88
Bulb 200	32	16	18	33	33	26.4 **±** 3.85**	41.33
Bulb 400	10	13	10	14	8	11 **±** 1.09***	75.56
Root 200	21	33	40	28	44	33.2 **±** 4.12	26.22
Root 400	20	16	12	10	22	16 **±** 2.28***	64.44

*Note:* All values are Mean ± SEM and statistically analyzed using One‐Way Analysis of Variance (ANOVA) followed by Dunnett’s multiple comparison test, *n* = 5. *p < .05, **p < .01, ***p < .001 when compared with control.

#### 4.4.2 Tail immersion method

The extracts administered at 200 and 400 mg/kg body weight demonstrated varying degrees of elevation in pain reaction time (PRT), percentage elongation of latency, and percentage of maximal possible effect (%MPE) in comparison to the control group, with the effect becoming more pronounced with increasing doses. Conversely, the standard substance, morphine sulfate (10 mg/kg), markedly intensified these measures. The anti‐nociceptive effect of the ethyl acetate fraction of the bulb of *C. suaveolens* at the 400 mg/kg dose level was found to be significant (*p* < .001) with 32.13% elongation of reaction time at 30 min and *p* < .01 with 23.03% elongation of reaction time at 60 min in the tail immersion model. Again, a significant result (*p* < .001) with 27.22% elongation of reaction time at 60 min is shown by the root of *C. suaveolens* at the 400 mg/kg dose level in the tail immersion model. This suggests that the extracts have central analgesic activity, which may be due to their effects on the spinal cord or brain. The delayed onset of action of the root extract compared to the bulb extract may be due to differences in the active compounds or their pharmacokinetics. Table 4a displays the findings for PRT and %MPE, while Table 4b exhibits the percentages of elongation of latency.

**TABLE 4a:** Effect of *Coelogyne suaveolens* bulb and root extract on reaction time and %MPE.

Test samples	Dose (mg/kg)	Pretreatment	Reaction times in seconds (mean ± SEM) and %MPE			
			30 min	60 min	90 min	120 min
Control	10 mL/kg	2.42 ± 0.07	2.45 ± 0.05 (0.24%)	2.54 ± 0.07 (0.95%)	2.48 ± 0.44 (0.48%)	2.44 ± 0.03 (0.16%)
Standard	50	2.55 ± 0.13	6.32 ± 0.07*** (30.28%)	8.22 ± 0.11*** (45.54%)	8.03 ± 0.07*** (44.01%)	7.82 ± 0.16*** (42.33%)
Bulb	200	2.46 ± 0.03	2.52 ± 0.04 (0.48%)	2.59 ± 0.01 (1.04%)	2.50 ± 0.07 (0.32%)	2.46 ± 0.06 (0)
Bulb	400	2.43 ± 0.01	3.61 ± 0.12*** (9.39%)	3.30 ± 0.10** (6.92%)	2.48 ± 0.24 (0.40%)	2.45 ± 0.21 (0.16%)
Root	200	2.43 ± 0.14	2.50 ± 0.10 (0.56%)	2.56 ± 0.10 (1.03%)	2.49 ± 0.13 (0.48%)	2.45 ± 0.12 (0.16%)
Root	400	2.38 ± 0.01	2.48 ± 0.11 (0.79%)	3.49 ± 0.11*** (8.80%)	2.91 ± 0.35 (4.20%)	2.51 ± 0.08 (1.03%)

*Note:* All values are Mean ± SEM and statistically analyzed using One‐Way Analysis of Variance (ANOVA) followed by Dunnett’s multiple comparison test, *n* = 5. *p < .05, **p < .01, ***p < .001 when compared with control.

**TABLE 4b:** Effect of *Coelogyne suaveolens* bulb and root extract on %elongation of latency time.

Test samples	Dose (mg/kg)	%elongation of latency time			
		30 min (%)	60 min (%)	90 min (%)	120 min (%)
Standard	50	61.23	69.10	69.12	68.80
Bulb	200	2.78	1.93	0.80	0.81
Bulb	400	32.13	23.03	0	0.41
Root	200	2.00	0.78	0.40	0.41
Root	400	1.21	27.22	14.78	2.79

#### 4.4.3 Hot plate method

*C. suaveolens* bulb and root extract at doses of 200 mg/kg and 400 mg/kg showed analgesic activity by the hot plate method. The bulb extract at 400 mg/kg showed significant activity (*p* < .01) at 30 min and (*p* < .001) at 60 min when compared to the control. Additionally, root extract at 400 mg/kg showed significant analgesic activity (*p* < .05) at 60 min. Among the two doses of *C. suaveolens*, the 200 mg/kg dose failed to show statistically significant analgesic activity when compared with the control. Furthermore, bulb extract at a dose of 400 mg/kg showed a 20.32%, 27.39%, and 2.56% increase in reaction time at 30, 60, and 90 min, respectively, whereas for root extract, the values are 10.92%, 9.96%, and −2.32%, which are demonstrated in Table 5.

**TABLE 5:** Changes in reaction time produced by *Coelogyne suaveolens using* the hot plate method.

Groups	Before treatment	After treatment			% increase in reaction time		
		30 min	60 min	90 min	30 min	60 min	90 min
Control	4.168 ± 0.499	4.165 ± 0.053	4.155 ± 0.043	4.14 ± 0.046	–	–	–
Standard	4.21 ± 0.057	6.943 ± 0.32***	7.29 ± 0.031***	7.0 ± 0.045***	64.917	73.159	66.271
Bulb200	4.17 ± 0.051	4.156 ± 0.052	4.20 ± 0.023	3.998 ± 0.088	−0.336	0.719	−4.125
Bulb400	4.168 ± 0.048	5.015 ± 0.0096**	5.31 ± 0.015***	4.275 ± 0.2604	20.321	27.399	2.567
Root200	4.185 ± 0.023	4.103 ± 0.0665	4.123 ± 0.036	4.088 ± 0.046	−1.959	−1.481	−2.318
Root400	4.165 ± 0.44	4.62 ± 0.091	4.58 ± 0.206*	4.068 ± 0.122	10.924	9.964	−2.329

*Note:* All values are Mean ± SEM and statistically analyzed using One‐Way Analysis of Variance (ANOVA) followed by Dunnett’s multiple comparison test, *n* = 5. *p < .05, **p < .01, ***p < .001 when compared with control.

### 4.5 Anxiolytic profiling

#### 4.5.1 Elevated plus maze method

EPM or elevated plus maze method is a well‐established test for evaluating the anxiolytic activity of compounds; it measures exploratory behavior in an anxiety‐provoking situation and is used to assess psychomotor capabilities and emotional aspects in rodents. This study examined the anxiolytic activity of the ethyl acetate fraction of the acetonic extract of *C. suaveolens* bulb and root. The results in Table 6 showed that the ethyl acetate fraction of bulb and root at a dose of 400 mg/kg body weight spent 5.00 and 5.20 s, respectively, in the open arm of the EPM apparatus, whereas in the standard it was 57.72 s, but in the control group it was 0.50 s. Again, it spent more time in the closed arm (279 and 268.8 s) in comparison to the standard (214 s). The other two doses (200 mg/kg) of bulb and root did not show any significant results. Moreover, all the extracts spent more time in the closed arm and less time in the open arm of the EPM apparatus in comparison to the standard, but in comparison to the control, they spent more time in the open arm and less time in the closed arm. From the observed result, it could be concluded that bulb 400 and root 400 have moderate anxiolytic activity.

**TABLE 6:** Evaluation of anxiolytic activity of *Coelogyne suaveolens* bulb and root by using the Elevated Plus Maze Method.

Test samples	Dose (mg/kg)	Open arm (OA)		Closed arm (CA)	
		Time spent (sec)	No. of entries	Time spent (sec)	No. of entries
Control	10 mL/kg	0.50 ± 0.32	0.80 ± 0.37	296 ± 1.87	14.60 ± 1.44
Standard	1	52.72 ± 0.86***	8.60 ± 0.51***	214 ± 1.08***	2.80 ± 0.37***
Bulb	200	2.40 ± 1.29	0.80 ± 0.37	294 ± 2.08	10.20 ± 1.20*
Bulb	400	5.00 ± 2.07*	1.00 ± 0.32	279 ± 2.41***	8.00 ± 0.84***
Root	200	2.80 ± 1.16	1.00 ± 0.32	292 ± 2.43	7.20 ± 0.86***
Root	400	5.20 ± 0.84*	2.40 ± 0.25	268.8 ± 1.88***	5.40 ± 0.51***

*Note:* All values are Mean ± SEM and statistically analyzed using One‐Way Analysis of Variance (ANOVA) followed by Dunnett’s multiple comparison test, *n* = 5. *p < .05, **p < .01, ***p < .001 when compared with control.

#### 4.5.2 Hole‐board test

In the hole board test, it has been observed that the ethyl acetate fraction of the root extract of *C. suaveolens* at doses of 400 mg/kg showed highly significant (*p* < .001) anxiolytic activity (41.40 head dipping) compared to the control. On the other hand, the number of heads dipped in the positive control group was 42.20, as depicted in Table 7. The other doses of the extract did not show any significant change compared to the control. This indicates that the root extract of *C. suaveolens* at doses of 400 mg/kg may have a more selective anxiolytic effect on certain aspects of behavior.

**TABLE 7:** Effect of *Coelogyne suaveolens* bulb and root extract on anxiolytic profiling using the Hole‐board test method.

Group	Dose (mg/kg)	Frequency of head‐dipping					Mean ± SEM
		M‐1	M‐2	M‐3	M‐4	M‐5	
Control	10 mL/kg	32	23	33	25	20	26.60 ± 2.54
Standard	1	45	39	46	40	41	42.20 ± 1.39***
Bulb	200	27	32	19	25	27	26.00 ± 2.10
Bulb	400	37	24	38	27	28	30.80 ± 2.82
Root	200	28	39	24	34	28	30.60 ± 2.68
Root	400	46	46	32	40	43	41.40 ± 2.60***

*Note:* All values are Mean ± SEM and statistically analyzed using One‐Way Analysis of Variance (ANOVA) followed by Dunnett’s multiple comparison test, *n* = 5. *p < .05, **p < .01, ***p < .001 when compared with control.

### 4.6 Sedative profiling

#### 4.6.1 Open‐field test

Based on Open Field data, it appears that the ethyl acetate fraction of bulb and root at the dose of 400 mg/kg at 30 min (71.4 and 58.2 no. of movements, respectively) (*p* < .001) when compared to the control group (112.2 no. of movements) and the values are very close to the standard (73 no. of movements), suggesting that the extract might have a potential sedative effect. The other two doses of 200 mg/kg of bulb and root extracts of the plant also showed reduced numbers of movements at 30 min (79.6 no. of movements) (*p* < .01) and (68.2 no. of movements) (*p* < .001), respectively. The extract may have a sedative effect, as the open field test showed a decline in locomotor activity and rearing behavior, as shown in Table 8.

**TABLE 8:** Effect of *Coelogyne suaveolens* bulb and root extract on the number of movements using the open field method.

Test samples	Dose (mg/kg)	Number of movements		
		0 min	30 min	60 min
Control	10 mL/kg	100.2 ± 13.56	112.2 ± 5.83	45.2 ± 8.53
Diazepam	1	86.4 ± 5.12	73 ± 2.92***	38 ± 1.95
Bulb	200	78.4 ± 13.7	79.6 ± 11.42**	60 ± 12.01
Bulb	400	72.2 ± 6.16	71.4 ± 5.33***	58.2 ± 7.55
Root	200	72.6 ± 4.86	68.2 ± 1.11***	39.4 ± 3.88
Root	400	72 ± 9.6	58.2 ± 5.83***	58 ± 3.44

*Note:* All values are Mean ± SEM and statistically analyzed using One‐Way Analysis of Variance (ANOVA) followed by Dunnett’s multiple comparison test, *n* = 5. *p < .05, **p < .01, ***p < .001 when compared with control.

#### 4.6.2 Hole cross method

During the hole cross test, there was a progressive decrease in the frequency with which the mice traversed between the chambers over a span of 2 h, as indicated in Table 9. In the hole cross method, it has been observed that in the ethyl acetate fraction of bulb and root at doses of 200 and 400 mg/kg body weight, the decreased locomotor activity of the experimented animals occurred. Among these samples, the ethyl acetate fraction of the root of *C. suaveolens* at doses of 200 and 400 mg/kg body weight mostly decreased the frequency of movement of the mice through the hole at 30 min (2.60 and 2.00) (*p* < .001) compared to standard (6.00) at 30 min. It was also observed that the ethyl acetate fraction of the bulb of the plant at a dose of 400 mg/kg body weight decreased the locomotor activity of the test animals at 30 min (5.60; *p* < .05). This result indicates that the extracts can also decrease exploratory behavior, which suggests a sedative effect.

**TABLE 9:** Effect of *Coelogyne suaveolens* bulb and root extract on the hole cross test.

Test samples	Dose (mg/kg)	Number of movements		
		0 min	30 min	60 min
Control	10 mL/kg	11.00 ± 0.44	8.40 ± 0.24	4.40 ± 0.81
Standard	1	7.60 ± 0.40*	6.00 ± 0.32	2.80 ± 0.58
Bulb	200	10.40 ± 1.81	7.60 ± 0.24	8.40 ± 2.11
Bulb	400	8.20 ± 0.49	5.60 ± 0.51*	7.80 ± 0.37
Root	200	8.00 ± 0.71	2.60 ± 1.21***	5.80 ± 0.37
Root	400	8.40 ± 0.51	2.00 ± 0.77***	5.60 ± 0.81

*Note:* All values are Mean ± SEM and statistically analyzed using One‐Way Analysis of Variance (ANOVA) followed by Dunnett’s multiple comparison test, *n* = 5. *p < .05, **p < .01, ***p < .001 when compared with control.

#### 4.6.3 Rotarod method

In the rotarod test, root extract at 400 mg/kg showed a significant decrease in the time spent on the rod (130 s at 30 min, 126 s at 60 min, and 134.60 s at 90 min), which, when compared to the control, was statistically significant (*p* < .001), as shown in Table 10. Again, bulb extract at 400 mg/kg showed a significant decrease in time spent on the rod (154 s at 30 min, 150 s at 60 min, and 157.4 s at 90 min); this also differed from the control in a statistically significant way (*p* < .001). The other two doses (200 mg/kg) of the root and bulb of the plant did not show any significant results. The rotarod test revealed a decrease in motor coordination and balance, which is a common effect of CNS depressants. Diazepam, on the other hand, was discovered to be a more effective muscle relaxant than the extracts.

**TABLE 10:** Effect of *Coelogyne suaveolens* bulb and root extract on motor coordination using the rota‐rod method.

Test samples	Dose (mg/kg)	Time (sec) of animals remained without falling from the rod at 24 rpm		
		30 min	60 min	90 min
Control	10 mL/kg	179.20 ± 0.80	180	180
Standard	1	76.40 ± 2.09***	115.4 ± 2.06***	156.4 ± 1.57***
Bulb	200	175.60 ± 2.04	174.4 ± 1.86	177.4 ± 1.67
Bulb	400	154 ± 3.05***	150 ± 3.91***	157.4 ± 2.54***
Root	200	176 ± 2.48	173.8 ± 1.96	176 ± 1.82
Root	400	130 ± 7.20***	126 ± 2.72***	134.6 ± 6.71***

*Note:* All values are Mean ± SEM and statistically analyzed using One‐Way Analysis of Variance (ANOVA) followed by Dunnett’s multiple comparison test, *n* = 5. *p < .05, **p < .01, ***p < .001 when compared with control.

### 4.7 Molecular docking analysis

Tables 11a and 11b as well as Figures 3a and 3b display the docking score and the non‐bond interactions of the docked compounds for analgesic activity. Our molecular docking analysis showed that every compound examined interacted with each of the target proteins. In terms of binding affinity toward COX‐1 (PDB ID: 5wbe), alpha‐linolenic acid (−5.9 kcal/mol), {bicyclo[3.1.1]heptan‐3‐one, 2,6,6‐trimethyl‐, (1.alpha., 2.beta., 5.alpha.)‐} (−5.8 kcal/mol), and geranyl acetate (−5.7 kcal/mol) were shown to be the strongest. The maximum binding affinity was shown by 3,6‐dimethyl‐2,3,3a,4,5,7a‐hexahydrobenzofuran with human COX‐2 (PDB ID: 5ikq). Several other compounds also exhibited strong binding affinity toward the target protein, including {bicyclo[3.1.1]heptan‐3‐one,2,6‐trimethyl‐, (1.alpha.,2.beta.,5.alpha.)‐} (−6.2 kcal/mol), geranyl acetate (−6 kcal/mol), and alpha‐linolenic acid (−6 Kcal/mol).

**TABLE 11a:** Binding affinity and interaction of the selected compounds with the amino acid residue of cyclooxygenase‐1 (PDB ID: 5wbe).

Compound name	Binding affinity (kcal/Mol)	Hydrogen bonds		Hydrophobic bonds	
		Conventional	Carbon‐hydrogen	Pi‐alkyl	Alkyl
1,6‐Octadien‐3‐ol,3,7‐dimethyl‐	−4.8			TRP100	ARG120, LEU112 (2), VAL119 (2), VAL116 (2), LEU357, LEU93 (2), LEU115 (2), ILE89
Bicyclo[3.1.1]heptan‐3‐one,2,6,6‐trimethyl‐, (1.alpha.,2.beta.,5.alpha.)‐	−5.8	ALA527	ILE523, GLY526	TYR355	LEU352 (2), ALA527 (3), VAL349, ILE523, VAL349 (2)
Isopulegol	−5.1	ARG120			ILE89 (3), VAL119 (2), LEU115, VAL116, PRO86 (2)
3,6‐Dimethyl‐2,3,3a,4,5,7a‐hexahydrobenzofuran	−5.2	ARG120 (2)			ILE89, LEU93 (2), LEU112 (2), LEU115 (3), VAL116 (3), VAL119, LEU357
Geranyl acetate	−5.7	ARG120	VAL116	TRP100	ILE89 (2), LEU115, LEU112, LEU92 (2), LEU93 (2)
9,12‐Octadecadienoic acid (Z,Z)‐	−5.1	ARG83			VAL116 (2), VAL119 (2), LEU115 (3), ILE89 (2), LEU93, LEU112 (2)
**Alpha‐linolenic acid**	**−5.9**	**ARG120 (2)**		**TRP100 (2)**	**VAL116, VAL119, LEU115 (2), LEU93 (2), LEU112 (2), LEU92, VAL103**

*Note*: Bold letters indicate the best binding score.

**TABLE 11b:** Binding affinity and interaction of the selected compounds with the amino acid residue of human cyclooxygenase‐2 (PDB ID: 5ikq).

Compound name	Binding affinity (kcal/Mol)	Hydrogen bonds		Hydrophobic bonds	
		Conventional	Carbon‐hydrogen	Pi‐alkyl	Alkyl
1,6‐Octadien‐3‐ol,3,7‐dimethyl‐	−5.8	TYR385		PHE381, TYR385, PHE518	VAL523, ALA527, LEU384, LEU352 (3), VAL523 (2), VAL349
Bicyclo[3.1.1]heptan‐3‐one,2,6,6‐trimethyl‐, (1.alpha.,2.beta.,5.alpha.)‐	−6.2	SER530	SER530		VAL349, ALA527, VAL523 (2), LEU352 (3)
Isopulegol	−5.6	ALA199		HIS207, TYR385 (2), TRP387 (2)	ALA202, LEU391, LEU390
**3,6‐Dimethyl‐2,3,3a,4,5,7a‐hexahydrobenzofuran**	**−6.8**			**TYR385, PHE518**	**VAL349 (2), LEU352 (3), VAL523 (2), ALA527 (2)**
Geranyl acetate	−6		HIS386	TYR385, HIS207, PHE200	LEU391 (2), LEU390, ALA202, ALA199 (2)
9,12‐Octadecadienoic acid (Z,Z)‐	−5.6			HIS207	ALA202, VAL444, VAL447, LEU294, LEU391 (2), LEU390
Alpha‐linolenic acid	−6		HIS207	HIS388, PHE395, TYR404	ALA202, VAL444, VAL447 (2), LEU391 (3), LEU390, VAL295

*Note*: Bold letters indicate the best binding score.

**FIGURE 3a:**
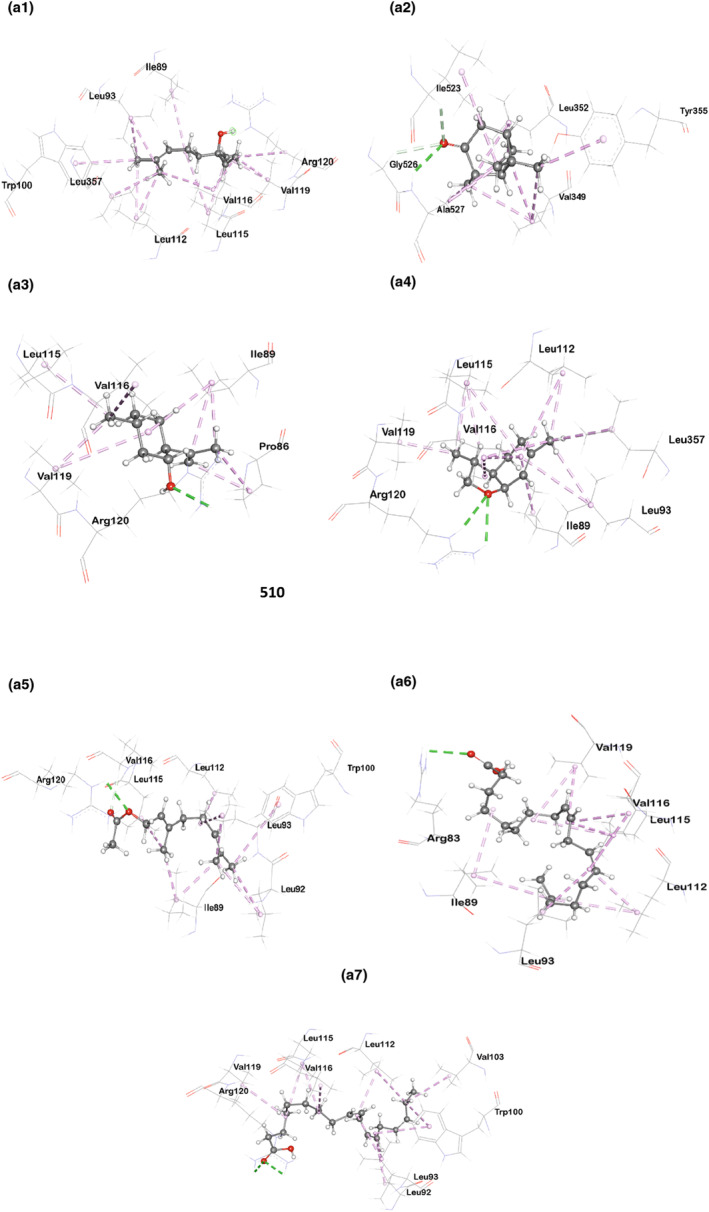
Molecular docking interaction of compounds against Cyclooxygenase‐1 (PDB ID: 5wbe): (a1) 1,6‐Octadien‐3‐ol,3,7‐dimethyl‐, (a2) Bicyclo[3.1.1]heptan‐3‐one,2,6,6‐trimethyl‐, (1.alpha.,2.beta.,5.alpha.)‐, (a3) Isopulegol, (a4) 3,6‐Dimethyl‐2,3,3a,4,5,7a‐hexahydrobenzofuran, (a5) Geranyl Acetate, (a6) 9,12‐Octadecadienoic acid (Z,Z)‐, and (a7) Alpha Linolenic Acid.

**FIGURE 3b:**
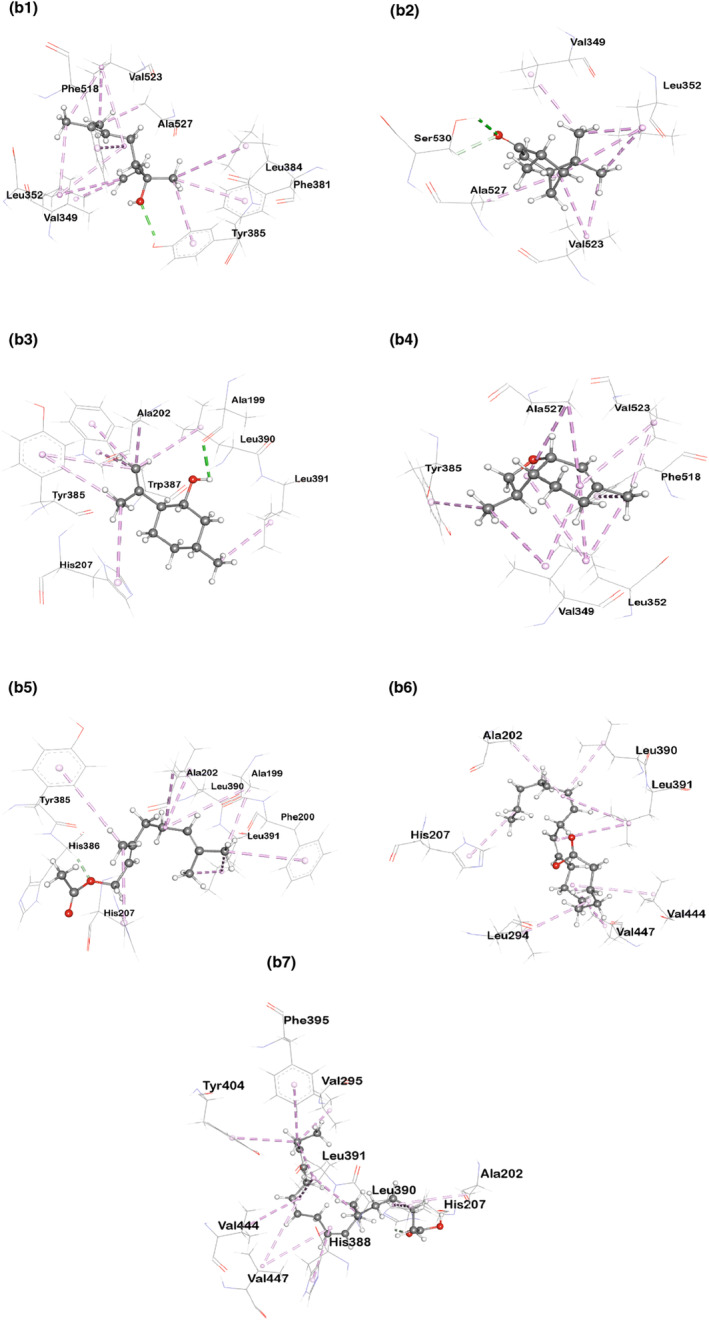
Molecular docking interaction of compounds against Cyclooxygenase‐2 (PDB ID: 5ikq): (b1) 1,6‐Octadien‐3‐ol,3,7‐dimethyl‐, (b2) Bicyclo[3.1.1]heptan‐3‐one,2,6,6‐trimethyl‐, (1.alpha.,2.beta.,5.alpha.)‐, (b3) Isopulegol, (b4) 3,6‐Dimethyl‐2,3,3a,4,5,7a‐hexahydrobenzofuran, (b5) Geranyl Acetate, (b6) 9,12‐Octadecadienoic acid (Z,Z)‐, and (b7) Alpha Linolenic Acid.

## 5 DISCUSSION

In this study, we have tested *C. suaveolens* bulb and root extract for identifying bioactive compounds and tested rodents for CNS, analgesic, and sedative profiling.

The acetic acid induction test is widely used to determine peripheral analgesic efficacy (Trongsakul et al., 2003). The pain induced by acetic acid occurs through an indirect mechanism involving the elevation of PG2 and PG2α levels at receptor sites within organ cavities. This suggests that carboxylic acid functions by indirectly enhancing the release of endogenous mediators (Ezeja et al., 2011; Lee & Choi, 2008). Acetic acid causes squirming in experimental animals via a chemosensitive nociceptor (Onasanwo & Elegbe, 2006). Nonsteroidal anti‐inflammatory medications work by inhibiting sensory neuron activation in response to inflammatory mediators (Akindele et al., 2011). Furthermore, the percentage reduction in the number of stomach squirms might indicate the extent of analgesia (Marchioro et al., 2005). The bulb and root extract, administered at 200 and 400 mg/kg doses, significantly reduced the average number of writhes, similar to the effect of standard aceclofenac. The idea behind the tail immersion method is that substances resembling morphine can specifically prolong the time it takes for the tail withdrawal reflex to occur in mice. The effectiveness of the extract's analgesic properties is gauged by the extension of the initial latency period (Elisabetsky et al., 1995; Pal et al., 1999). Both the root and bulb extracts, specifically at a 400 mg/kg dose, increased the reaction time, % MPE (maximum possible effect), and basal latency. The effect of the bulb extract at this dose remained stable between 30 and 60 min. The delayed onset of action of the root extract, as compared to the bulb extract, might be attributed to differences in active compounds or their pharmacokinetics.

The fact that the extracts enhance basal latency suggests that they may activate a centrally mediated analgesic mechanism (Bachhav et al., 2009). Sensory nerves sensitize the nociceptors in this approach, and the participation of endogenous chemicals such as prostaglandins is minimized (Uche & Aprioku, 2008). The hot plate test is commonly used for evaluating centrally acting analgesics, but it may not effectively assess peripherally acting analgesics. It has limitations, as sedatives, muscle relaxants, and psychotomimetics can produce false positives, and mixed opiate agonists‐antagonists yield unreliable results. However, *C. suaveolens* bulb extract at 400 mg/kg demonstrated significant activity at 30 min (*p* < .01) and 60 min (*p* < .001) compared to the control. This suggests that *C. suaveolens* exhibits both central and peripheral analgesic activity, as indicated by the results from these three methods.

The EPM is an in vivo method used to evaluate the potential for anxiolytic activity in experimental animals. In this test, animals typically exhibit a preference for the enclosed areas of the maze and may avoid open segments that lack protective walls, which are considered less favorable (Collimore & Rector, 2014).

The manifestation of notable alterations in the open‐arm behavior of animals subjected to experimental plant extracts can be interpreted as indicative of the anxiolytic efficacy of these extracts. An established anxiolytic drug, like benzodiazepine, is commonly used for this purpose. In the CNS (central nervous system), GABA (gamma‐aminobutyric acid) plays a crucial role as a major neurochemical. Plant extracts may exert their effects by enhancing GABAergic inhibition in the CNS, leading to hyperpolarization and a reduced firing rate of vital neurons in the brain. Alternatively, they may directly activate GABA receptors (Verma et al., 2010). Previous research suggests that plants containing saponins, tannins, and flavonoids may have an impact on various CNS disorders (Yadav et al., 2013). Initial phytochemical analyses and evaluations of plant extracts have suggested that neuroactive steroids and flavonoids might operate as agents binding to GABA receptors within the central nervous system, akin to molecules resembling benzodiazepines (Verma et al., 2010). When compared to the control group in the current investigation, the bulb and root extracts at 400 mg/kg showed mild anxiolytic efficacy.

The hole board test is an alternative method for assessing anxiolytic activity. It depends on tracking experimental animals' head‐dipping behavior while utilizing the hole‐board instrument. If the animals are sensitive to changes in their emotional or anxiolytic state, an increase in head‐dipping behavior might be observed (Foyet et al., 2012; Wei et al., 2007). Another crucial aspect of assessing a drug's impact on the central nervous system (CNS) is to observe its effect on the locomotor activity of the test animals. Locomotor activity can indicate CNS stimulation, where increased alertness leads to heightened motion activity, or it can imply a sedative effect when motion activity is reduced (Bhattacharya & Satyan, 1997). The level of CNS excitement can be gauged from locomotor activity, and a decrease in this activity is closely associated with CNS depression‐induced sedation (Islam et al., 2015). In this study, the CNS depressant activity of the ethyl acetate fraction of acetonic extract from *C. suaveolens* bulb and root was evaluated using three behavioral methods. The open field method measured the animals’ locomotor activity, providing insights into the general level of CNS activity. The hole cross method assessed exploratory behavior, which is also related to CNS activity. Lastly, the Rotarod method evaluated the extract's ability to impair motor coordination and balance in animals. All three methods indicated that the extract may possess potent sedative activity.

Molecular docking is commonly used to predict the binding orientation of small molecules to enzymes and receptors (Gohlke et al., 2000). Cyclooxygenase‐1 (COX‐1) and cyclooxygenase‐2 (COX‐2) enzymes play pivotal roles in prostaglandin synthesis and have been identified as potential therapeutic targets for various inflammatory and pain‐related conditions. Our analysis revealed significant insights into the binding interactions of the compounds with COX‐1 (PDB ID: 5wbe) and COX‐2 (PDB ID: 5ikq). Among the compounds investigated, some of them, like {1,6‐octadien‐3‐ol, 3,7‐dimethyl‐}, isopulegol, and geranyl acetate, revealed analgesic behaviors (Nazimova et al., 2016; Peana et al., 2003 Quintans‐Júnior et al., 2013), while alpha‐linolenic acid, {bicyclo[3.1.1]heptan‐3‐one, 2,6,6‐trimethyl‐, (1.alpha.,2.beta.,5.alpha.)‐}, and {3,6‐dimethyl‐2,3,3a,4,5,7a‐hexahydrobenzofuran} have been found to possess anti‐inflammatory activity from previous studies (Chou et al., 2018; Miao et al., 2019; Zhao et al., 2005).

In our present study, all the compounds showed a favorable docking score toward both cyclooxygenase‐1 (PDB ID: 5wbe) and human cyclooxygenase‐2 (PDB ID: 5ikq), with alpha‐linolenic acid being the highest (binding affinity: −5.9) for cyclooxygenase‐1 and 3,6‐dimethyl‐2,3,3a,4,5,7a‐hexahydrobenzofuran (binding affinity: −6.8) for cyclooxygenase‐2. Besides, bicyclo[3.1.1]heptan‐3‐one,2,6,6‐trimethyl‐, (1.alpha.,2.beta.,5.alpha.)‐, and geranyl acetate also displayed a noteworthy affinity for cyclooxygenase‐1, with docking scores of −5.8 and −5.7, respectively, where it is −6.2 and −6 for cyclooxygenase‐2, respectively. These high docking scores suggest strong binding interactions between these compounds and the respective enzymes, indicating their potential as potent inhibitors. Interestingly, against Cyclooxenase‐1, all the examined compounds interacted with ARG120 residue either by forming hydrogen or hydrophobic bonds except {bicyclo[3.1.1]heptan‐3‐one, 2,6,6‐trimethyl‐, (1.alpha.,2.beta.,5.alpha.)‐} and {9,12‐octadecadienoic acid (Z,Z)‐}. However, {bicyclo[3.1.1]heptan‐3‐one, 2,6,6‐trimethyl‐, (1.alpha.,2.beta.,5.alpha.)‐} formed a hydrophobic bond with TYR355 residue. These interactions with ARG120 and TYR355 are notable, as both residues play a significant role in regulating the entry of substrates or inhibitors into the long hydrophobic channel of cyclooxygenases (Cingolani et al., 2017).

As for Cyclooxygenase‐2, {3,6‐dimethyl‐2,3,3a,4,5,7a‐hexahydrobenzofuran}, geranyl acetate, isopulegol, and {1,6‐octadien‐3‐ol,3,7‐dimethyl‐} showed hydrophobic interactions with TYR385 residue. {1,6‐Octadien‐3‐ol,3,7‐dimethyl‐} also formed a conventional hydrogen bond with the TYR385 residue. This interaction is particularly significant due to the fact that TYR385 is known to play a vital role in the transformation of arachidonic acid into prostaglandins. TYR385 is responsible for commencing the oxygenation of arachidonic acid in cyclooxygenases by abstracting the pro‐S hydrogen at the C‐13 position, which is an important step in the conversion (Schneider & Brash, 2000). Again, {bicyclo[3.1.1]heptan‐3‐one, 2,6,6‐trimethyl‐, (1.alpha.,2.beta.,5.alpha.)‐} forms two hydrogen bonds (conventional and carbon‐hydrogen) with SER530, which is also significant as binding with SER530 may change the catalytic activity of Cyclooxygenase‐2 (Giménez‐Bastida et al., 2019).

These findings suggest that our compounds effectively target the active site residues of Cyclooxygenase‐1 and Cyclooxygenase‐2, suggesting their potential to disrupt the enzymatic activity and inhibit prostaglandin synthesis.

## 6 CONCLUSION

These investigations unveil promising pharmacological possibilities that could contribute to the advancement of modern medicine in deriving drugs from plant origins, offering diverse therapeutic potentials with minimal adverse effects. *C. suaveolens* emerges as a potential remedy for certain neuropharmacological conditions and pain relief. This research holds the potential to shed light on crucial domains within biomedical science that have yet to be extensively explored by researchers. In addition, seven compounds’ binding orientations to the cyclooxygenase‐1 and cyclooxygenase‐2 enzymes were explored in our molecular docking investigation. Alpha‐linolenic acid, 3,6‐dimethyl‐2,3,3a,4,5,7a‐hexahydrobenzofuran, {Bicyclo[3.1.1]heptan‐3‐one,2,6,6‐trimethyl‐, (1.alpha.,2.beta.,5.alpha.)}, and geranyl acetate were among the compounds tested, and their promising docking scores for both enzymes suggested that they could be able to block prostaglandin synthesis. The compounds’ capacity to target key regions involved in the enzymatic activity is further demonstrated by their interactions with important residues, such as ARG120 and TYR355 in cyclooxygenase‐1 and TYR385 and SER530 in cyclooxygenase‐2. These results provide further evidence that the compounds under study have the potential to operate as analgesic drugs by inhibiting the enzymatic activity of cyclooxygenases 1 and 2. In a nutshell, the investigation into the medicinal potential of *C. suaveolens* has yielded promising results, but several uncertainties warrant consideration in future research. First, small sample sizes and replication across experiments raise concerns about statistical robustness. Future studies should incorporate larger sample sizes and multiple replicates to enhance the validity of results. Additionally, while dose‐dependent effects were noted in some experiments, a more comprehensive dose–response analysis is needed to establish the optimal therapeutic dosage. Furthermore, it is crucial to acknowledge that the observed effects in Swiss albino mice may not directly translate to human responses, necessitating further research using diverse animal models and, ultimately, clinical trials. Lastly, ongoing efforts to identify and characterize the exact bioactive compounds causing the therapeutic potential in the extract's ethyl acetate fraction should be pursued to provide a more comprehensive understanding of the plant's therapeutic potential.

## AUTHOR CONTRIBUTIONS

**Taslima Akter Eva:** Conceptualization (equal); data curation (equal); formal analysis (equal); investigation (equal); methodology (equal); software (equal); writing – original draft (lead); writing – review and editing (equal). **Husnum Mamurat:** Conceptualization (equal); data curation (equal); formal analysis (equal); software (equal); writing – review and editing (equal). **Md. Habibul Hasan Rahat:** Data curation (supporting); formal analysis (supporting); writing – original draft (supporting); writing – review and editing (supporting). **S. M. Moazzem Hossen:** Conceptualization (equal); data curation (equal); methodology (equal); project administration (equal); validation (lead).

## CONFLICT OF INTEREST STATEMENT

The authors state that the publication of this article does not present any conflicts of interest for them.

## INSTITUTIONAL REVIEW BOARD STATEMENT

The study protocol was authorized in accordance with government directives under Pharm/P&D/CUDP‐16,2023:10 by the Department of Pharmacy, University of Chittagong, Chittagong, Bangladesh.

## Data Availability

Upon reasonable request, the corresponding author will provide the data that back up the study's conclusions.
